# Divergent foraging strategies between populations of sympatric matrilineal killer whales

**DOI:** 10.1093/beheco/arad002

**Published:** 2023-03-04

**Authors:** Jennifer B Tennessen, Marla M Holt, Brianna M Wright, M Bradley Hanson, Candice K Emmons, Deborah A Giles, Jeffrey T Hogan, Sheila J Thornton, Volker B Deecke

**Affiliations:** Conservation Biology Division, Northwest Fisheries Science Center, National Marine Fisheries Service, National Oceanic and Atmospheric Administration, Seattle, WA 98112, USA; Lynker Technologies LLC, Leesburg, VA 20175, USA; Conservation Biology Division, Northwest Fisheries Science Center, National Marine Fisheries Service, National Oceanic and Atmospheric Administration, Seattle, WA 98112, USA; Pacific Biological Station, Fisheries and Oceans Canada, Nanaimo, BC V9T 6N7, Canada; Conservation Biology Division, Northwest Fisheries Science Center, National Marine Fisheries Service, National Oceanic and Atmospheric Administration, Seattle, WA 98112, USA; Conservation Biology Division, Northwest Fisheries Science Center, National Marine Fisheries Service, National Oceanic and Atmospheric Administration, Seattle, WA 98112, USA; Department of Wildlife, Fish, & Conservation Biology, University of California, Davis, Davis, CA 95616, USA; Cascadia Research Collective, Olympia, WA 98501, USA; Pacific Science Enterprise Centre, Fisheries and Oceans Canada, West Vancouver, BC V7V 1N6, Canada; Institute of Science and Environment, University of Cumbria, Ambleside, Cumbria LA22 9BB, UK

**Keywords:** bio-logging DTAG, foraging behavior, Northern Resident killer whale, prey capture, Southern Resident killer whale

## Abstract

In cooperative species, human-induced rapid environmental change may threaten cost–benefit tradeoffs of group behavioral strategies that evolved in past environments. Capacity for behavioral flexibility can increase population viability in novel environments. Whether the partitioning of individual responsibilities within social groups is fixed or flexible across populations is poorly understood, despite its relevance for predicting responses to global change at the population and species levels and designing successful conservation programs. We leveraged bio-logging data from two populations of fish-eating killer whales (*Orcinus orca*) to quantify patterns of fine-scale foraging movements and their relationships with demography. We reveal striking interpopulation differences in patterns of individual foraging behavior. Females from the endangered Southern Resident (SRKW) population captured less prey and spent less time pursuing prey than SRKW males or Northern Resident (NRKW) females, whereas NRKW females captured more prey than NRKW males. The presence of a calf (≤3 years) reduced the number of prey captured by adult females from both populations, but disproportionately so for SRKW. SRKW adult males with a living mother captured more prey than those whose mother had died, whereas the opposite was true for NRKW adult males. Across populations, males foraged in deeper areas than females, and SRKW captured prey deeper than NRKW. These population-level differences in patterns of individual foraging behavior challenge the existing paradigm that females are the disproportionate foragers in gregarious resident killer whales, and demonstrate considerable variation in the foraging strategies across populations of an apex marine predator experiencing different environmental stressors.

## Teaser

Rapid environmental change may disrupt cost–benefit tradeoffs of foraging strategies in cooperative species, yet whether populations differ in the partitioning of individual responsibilities within social groups is poorly understood. By combining fine-scale movement and sound data from bio-logging tags and demographic data from long-term population censuses, we reveal opposite patterns of individual foraging behavior between killer whale populations with divergent population trajectories, demonstrating variation in the partitioning of foraging responsibilities in an apex marine predator.

## INTRODUCTION

Understanding the factors shaping group foraging is central to behavioral ecology. A group’s foraging strategies are optimized to balance the benefits of increased resource acquisition and reduced predation risk with the costs of intragroup competition and interference (Clark and Mangel 1984; Cvikel et al. 2015; Senior et al. 2016; Lihoreau et al. 2017; Ding et al. 2020). In gregarious species, individual foragers can optimize group foraging by partitioning responsibilities such as sharing information about quality foraging habitats, hunting, prey provisioning, and alloparental care (e.g., Boesch et al. 2006; Gero et al. 2009; Friedlaender et al. 2011; Wright et al. 2016). Integral to the partitioning of foraging roles is the inherent diversity in nutritional requirements among individuals within groups (Maklakov et al. 2008; Furrer et al. 2012), for example mixed-sex groups in sexually size-dimorphic species, such as killer whales, *Orcinus orca* (Miller et al. 2010). Sex differences in energetic requirements can affect fitness via both survival and reproduction (Maklakov et al. 2008), and therefore sexual segregation of foraging tasks or areas is a common resource maximizing strategy across size-dimorphic vertebrate species (Ruckstuhl and Neuhaus 2000; Bonenfant et al. 2004; Baird et al. 2005; Bearhop et al. 2006; Breed et al. 2006; Catry et al. 2006; Maklakov et al. 2008; Beerman et al. 2016; Photopoulou et al. 2020). Demographic patterns can also play an important role in shaping foraging responsibilities. For example, lactation is associated with considerable energetic costs (Williams et al. 2011; Christiansen et al. 2016), especially if migration occurs concurrently (e.g., Lockyer 2007; Christiansen et al. 2016). It may therefore be advantageous for lactating mothers to remain with nursing offspring and provide protection rather than expend energy on foraging (Lacy et al. 2017). Moreover, lactation can be time-intensive and can restrict a mother’s locomotory abilities (Videsen et al. 2017), thereby limiting the time and opportunities available for foraging. The presence of maternal kin can also shape foraging strategies. In species in which females have a prolonged post-reproductive phase, grandmothers have been shown to increase survival of offspring (Foster et al. 2012), in part due to prey-sharing, especially with their adult sons (Wright et al. 2016).

Environmental pressures, especially those from human-induced rapid environmental change, threaten cost–benefit tradeoffs of behavioral strategies that evolved in past environments (Sih et al. 2011). A defining characteristic of the Anthropocene is the unprecedented speed at which environments are changing (Steffen et al. 2011; Lewis and Maslin 2015). Under rapid change, previously optimal behaviors may be mismatched to present environmental conditions (Sih et al. 2011) and may become maladaptive (Merkle et al. 2022). Populations that have the capacity for behavioral flexibility in response to novel pressures, for example greater genetic variation or an evolved ability to be flexible in resource use, may have a greater chance of survival (Sih et al. 2011). In populations of gregarious species that engage in group behaviors with divisions of responsibilities, whether the partitioning of responsibilities is fixed across populations experiencing different stressors is poorly understood, despite the growing awareness that behavioral context shapes responses to environment (Ellison et al. 2012; DeRuiter et al. 2017; McHuron et al. 2017; Pirotta et al. 2018; Southall et al. 2019). Understanding behavior is critical for predicting wildlife responses to global change (Berger-Tal et al. 2011; Berger-Tal and Saltz 2016; Gil et al. 2020) and improves the success of species conservation programs (Blumstein and Fernández-Juricic 2010; Berger-Tal et al. 2011; Berger-Tal and Saltz 2016; Gil et al. 2020). Therefore, determining the factors driving the partitioning of individual responsibilities in group systems would advance an understanding of the capacity of gregarious species to cope with novel pressures, which could enhance conservation programs in the face of current and forecasted global declines in biodiversity.

We leverage a unique bio-logging data set that provides a rare opportunity to test whether the partitioning of foraging roles is consistent between sympatric populations of a social, apex marine predator that forage on the same prey species and display similar social structure, yet have experienced divergent population growth trajectories in recent years (Murray et al. 2021). Northern and Southern Resident killer whales (hereafter NRKW and SRKW, respectively) live in the coastal waters along the west coast of the United States and Canada, in the eastern North Pacific Ocean (Bigg 1982; Heimlich-Boran 1988; Ford et al. 1998). These populations are structured around matrilines, multiple groups of mature females and their philopatric offspring of both sexes, related to a common female ancestor (Bigg et al. 1990; Stredulinsky et al. 2021). Emigration between matrilines is extremely rare, emigration and immigration between NRKW and SRKW populations has never been documented, and both sexes remain with their natal matriline for life with rare exceptions (Bigg et al. 1990; Barrett-Lennard 2000; Ford et al. 2000, 2011, 2018; Parsons et al. 2009). The presence of a living mother increases the survival of her weaned offspring, particularly her adult sons (Foster et al. 2012) and the presence of living grandmothers, especially post-reproductive grandmothers, increases the survival of their grandoffspring (Nattrass et al. 2019).

The group foraging system of resident killer whales is characterized by group travel to foraging sites, individual pursuit and capture of prey followed by prey-sharing with group members (Wright et al. 2016). Specifically, groups are thought to employ collective ecological knowledge to travel to particular foraging areas as cohesive social units, and to search for and locate prey patches that group members then exploit individually. Individuals pursue prey, often simultaneously, and then share prey with one or more group members (Wright et al. 2016) as a critical and necessary mechanism for the group to meet its nutritional requirements. Therefore, the individual foraging behavior that begets prey consumption among group members is a fundamental component of the cooperative acquisition of nutrients that makes up the group foraging system in resident killer whales. Once-abundant and reliable stocks of salmon (*Oncorhynchu*s spp.), primarily Chinook (*O. tshawytscha*) and to a lesser extent chum (*O. keta*) and coho (*O. kisutch*; Ford and Ellis 2006; Hanson et al. 2010, 2021; Ford et al. 2016), make up the majority of NRKW and SRKW diets. Typically, salmon are initially chased, depending on the species either at the surface or at depth, and then captured and brought up to the surface (Wright et al. 2017; Tennessen et al. 2019a) where they are broken apart and shared (Ford and Ellis 2006; Wright et al. 2016). Research has revealed that NRKW mothers and post-reproductive grandmothers disproportionately share prey with their offspring and grandoffspring, and maternal sharing with adult offspring is predominantly directed toward males, to maximize inclusive fitness (Wright et al. 2016) as male reproductive success increases with age (Ford et al. 2011, 2018). While it has generally been assumed that the partitioning of foraging and provisioning roles among age/sex classes is fixed across populations of resident-ecotype killer whales, this has never been investigated, yet has important implications for advancing behavioral ecology theory and for developing effective conservation programs. While both NRKW and SRKW experienced declines during the 1960s and 1970s driven by the live-capture fishery for aquaria (Bigg and Wolman 1975; Reeves and Leatherwood 1984), their population trajectories have since diverged (Murray et al. 2021), despite their overlapping spatial distributions and similar ecology and prey preferences. Since annual population censuses began in 1973, NRKW, listed as Threatened under Canada’s Species at Risk Act (SARA; DFO 2017), have experienced nearly continuous growth, increasing by more than 50% of their 2001 population size between 2001 and 2017 (Towers et al. 2015; DFO 2020). In contrast, SRKW, listed as Endangered under both SARA and the US Endangered Species Act (NMFS 2016; DFO 2017), have exhibited virtually no net growth since population censuses began in 1976 (NMFS 2016). While the differences in causal mechanisms underlying the two population trajectories are not fully understood, models of cumulative impacts of primary threats including limited prey availability, vessel disturbance (both acoustic and physical) and exposure to persistent organic pollutants explain the observed population trends reasonably well (Murray et al. 2021).

The divergent population growth trajectories between these sympatric populations and their differing levels of environmental stressors including human-generated ambient noise, vessel traffic and legacy contaminants (NMFS 2016; DFO 2020), provide a rare opportunity to test whether individual foraging responsibilities in a gregarious species are fixed across populations. Here, we leverage fine-scale behavioral data from high-resolution, multisensor bio-logging tags attached to NRKW and SRKW killer whales, paired with demographic data from long-term population censuses, to understand the factors that promote diversity in foraging behavior. Specifically, we test 1) whether sex-based patterns of foraging behavior are consistent between NRKW and SRKW populations, despite divergent population growth trajectories (Murray et al. 2021), differences in population stressors (NMFS 2016; DFO 2020) and the potential for shifting cost–benefit tradeoffs of optimal foraging behavior strategies (Merkle et al. 2022). We furthermore test 2) whether foraging behavior is predicted by reproductive status and demography, given the costs of reproduction in long-lived mammalian species (Boness and Bowen 1996; Christiansen et al. 2016; Thometz et al. 2016; Miketa et al. 2018), the importance of post-reproductive females as leaders and prey provisioners in resident killer whale populations especially when prey are limited (Brent et al. 2015; Nattrass et al. 2019), and the benefit of a living mother on the survival likelihood of her adult sons (Foster et al. 2012).

## METHODS

Broadly, we attached multisensor bio-logging archival tags (Dtag versions 2 and 3, Johnson and Tyack 2003) by suction cup to NRKW and SRKW to quantify individual subsurface foraging behavior in their core summer habitats in the inland coastal waters of British Columbia, Canada and Washington, United States. Following tag recovery, we downloaded and processed data, computed foraging metrics, and constructed statistical models to compare individual foraging behavior between populations.

### Study design

**Figure 1:**
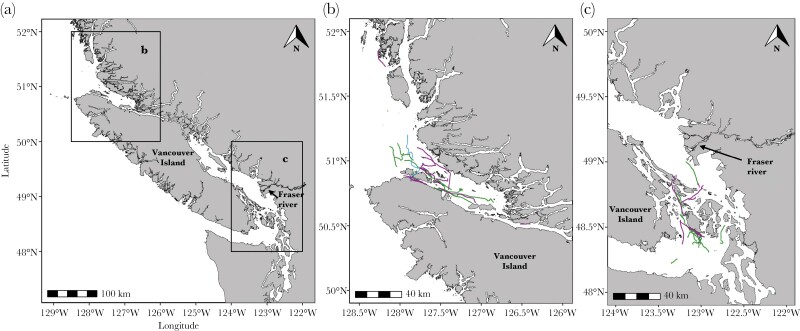
Map of study areas of Dtag deployments on resident killer whales in Washington State, United States and British Columbia, Canada (a). NRKW population (b) and SRKW population (c) were tagged on opposite ends of Vancouver Island (female = purple, male = green, unknown sex = blue).

NRKW were tagged in the Queen Charlotte Strait and Central Coast regions of British Columbia in August and September, between 2009 and 2012. SRKW were tagged in Haro Strait and the Straits of Georgia and Juan de Fuca around the San Juan Islands, Washington, in September in 2010, 2012, and 2014 (Figure 1). Tagging methodology is described elsewhere (NRKW: Wright et al. 2017; SRKW: Holt et al. 2017). Briefly, we identified individual killer whales using photo-ID catalogues based on unique natural markings on their dorsal fins and the gray areas immediately posterior to their dorsal fins (Bigg 1987). We applied Dtags at the base of the dorsal fin using a 7-m hand-held carbon fiber pole from the bow of a small research vessel. Thirty-four and twenty-three Dtags were attached to NRKW and SRKW, respectively. Individual reactions to tagging ranged from no response to flinching or diving, and all individuals returned to pretagging surfacing behavior within 5 min. Inspection of time series of subsurface diving behavior of tagged whales from NRKW and SRKW, generated from tag pressure sensor data during analysis (described below), indicated that individuals from both populations responded similarly to initial tagging and time to acclimation. Tags generally remained attached during daylight hours as programmed, although some fell off prematurely due to water flow, rubbing, or impactful behaviors at the surface (e.g., breaching). We tagged individuals opportunistically, ensuring whenever possible that we selected a balanced representation of age and sex classes. All tagged animals were at least 2 years old. One NRKW and two SRKW were tagged twice in different years. Dtags recorded depth, body orientation, and movement using triaxial accelerometers and magnetometers that sampled at 50–250 Hz, and sound using stereo hydrophones that sampled at 96–240 kHz (see details in Holt et al. 2017; Wright et al. 2017). We omitted five deployments: three NRKW tag deployments which fell off within minutes of deployment and were too short in duration to be calibrated, and two tag deployments (one from each population) for which the accelerometers malfunctioned.

We conducted focal follows of each tagged whale for the duration of the deployment as conditions allowed, during which we obtained periodic GPS fixes at surfacings (see details in Giles 2014; Wright et al. 2017) for subsequent track reconstruction (see *Data processing*). We also collected prey remains using fine-meshed dip nets (Ford and Ellis 2006; Hanson et al. 2010) to validate predation events and identify prey species by fish scale morphology or genetic analysis, and noted changes in tag orientation to aid data calibration. Prey species collected included Chinook and to a lesser extent chum and coho salmon, consistent with studies of diet composition of resident killer whales in the summer foraging habitat where our study was conducted (Ford et al. 1998, 2010, 2016; Ford and Ellis 2006; Hanson et al. 2010).

### Data processing

We used a VHF receiver to locate and recover tags following detachment. We downloaded the data and used the 2014 Dtag toolbox (www.soundtags.org/dtags/dtag-toolbox) in MATLAB v R2016b (The MathWorks, Natick, MA) to calibrate sound and movement data based on sensor characteristics and tag orientation on the whale (see details in Holt et al. 2017; Wright et al. 2017). Several time series of data were produced over the duration of the deployment: temperature-corrected depth, triaxial orientation (pitch, roll, and heading), triaxial acceleration, and ambient sound (Johnson and Tyack 2003).

For each deployment, we parsed data into individual dives using the “find_dives” function from the 2014 Dtag toolbox, by searching for depth excursions >1 m bounded by surfacings <0.5 m (see details in Tennessen et al. 2019a). We omitted dives <4 s in duration, as these likely resulted from incomplete surfacings or from fluctuations in the pressure sensor data due to a high sampling rate. We checked dives manually to ensure accuracy. For every dive, we computed start and end times, and excluded dives that began within the first 5 min of the onset of the deployment, to account for short-term behavioral responses to tagging. We visually inspected all dive profiles to confirm that 5 min was a conservative threshold for the duration of these behavioral responses.

To obtain animal tracks and align foraging behavior with bathymetry, we computed geo-referenced pseudotracks (hereafter “tracks”; for details, see Wright et al. 2017). Because the Dtags did not contain GPS sensors, we used the 2014 Dtag toolbox in MATLAB to dead-reckon the accelerometer and magnetometer data to create best estimates of tracks, by identifying the next position in time based on the previous position (Wilson et al. 2007). To constrain accumulated spatial error associated with drift, we forced the tracks through known GPS coordinates taken periodically when whales surfaced (Wilson et al. 2007), using the TrackReconstruction package in R (Battaile 2019; R Core Team 2020). We used bathymetry data from the GEBCO database (www.gebco.net; 15 arc-second resolution) and the marmap R package (Pante and Simon-Bouhet 2013) to compute bathymetry values for every dive, taken as the water depth at the estimated GPS location of the start of each dive. Differences in the magnitude of track error between deployments are presumed to have negligible impact on bathymetry estimates because these errors are unlikely to exceed the spatial resolution of the bathymetry data.

### Foraging metrics

Sounds of foraging activity detected in tag acoustic data can indicate prey pursuit and capture events (e.g., Holt et al. 2019; Wright et al. 2021). Excess noise in some audio recordings from water flowing over the tag or from nearby vessels prevented us from computing sound variables for some deployments. Therefore, we used kinematic detection of foraging behavior validated by available acoustic data to identify prey capture events, which is an established method that maximizes sample size in the absence of complete acoustic data (Allen et al. 2016; Tennessen et al. 2019a). We viewed spectrograms (512 point, Hann window, 50% overlap) alongside plots of depth and angle of arrival between the two hydrophone channels to identify all occurrences of foraging sounds: slow and fast echolocation clicks (interclick intervals of >100 and 11–100 ms, respectively, associated with searching for and pursuing prey), buzzes (interclick interval <11 ms, associated with final pursuit of prey) and prey handling sounds (those of crunching and tearing, produced during prey processing), and mapped these sounds to dives (see details in Holt et al. 2019). For movement data, we partitioned each dive into descent, bottom (≥70% of maximum dive depth) and ascent phases (Arranz et al. 2016; Tennessen et al. 2019a), and for each phase computed three variables previously demonstrated to predict prey capture (Tennessen et al. 2019a): jerk peak (maximum peak of the jerk signal, the rate of change of triaxial acceleration, adjusted by the median jerk signal, see Ydesen et al. 2014; Allen et al. 2016; Arranz et al. 2016; Tennessen et al. 2019a), roll at jerk peak (absolute value of the roll at the time of jerk peak, in degrees), and circular variance in heading using the “circ_var” function in the CircStat package (Berens 2009) in MATLAB.

We filtered prey capture dives from each deployment time series by setting minimum thresholds for jerk peak, roll, and heading variance determined from known prey capture dives identified using available acoustic data (see Supplementary Information, data presented in Supplementary Table S1). We determined these thresholds for each population separately by matching detection sensitivities between populations (true-positive rate of 100%, false-positive rate of 26%, for dives ≥50 m). Thus, the filter detected all acoustically confirmed prey capture dives, while minimizing the false-positive rate. When detecting prey capture events, there is a tradeoff between maximizing true positives (accuracy) and minimizing false positives (specificity). Since we were interested in quantifying prey capture in populations foraging under conditions of limited prey availability and accessibility (NMFS 2016; DFO 2017; Lacy et al. 2017), it was critical to maximize confidence in our ability to detect true prey captures (achieve a miss rate near zero) and accept the concomitant higher false-positive rate.

To quantify the total time a whale spent searching for prey during each deployment, we summed the duration of all dives during which only slow echolocation clicks were detected. While it is likely that some searching dives also included other foraging behaviors, such as pursuit and capture, this conservative approach allowed us to focus exclusively on those dives during which the individual was acoustically scanning the environment. Previous research suggests that this search phase is a distinct component of foraging behavior, which primarily occurs at the surface and is behaviorally differentiated from pursuit and prey capture phases (Tennessen et al. 2019b; Holt et al. 2021b). To quantify the proportion of time a whale engaged in prey capture, we summed the duration of dives that resulted in prey capture and divided this by the total deployment time. To quantify the proportion of time a whale engaged in travel or resting dives, we summed the duration of dives <30 m during which no echolocation clicks or sounds of prey capture or handling were produced (Holt et al. 2013; Noren and Hauser 2016) and divided this by the total deployment time.

We used photographic data gathered during population censuses conducted by the Center for Whale Research (SRKW) and Fisheries and Oceans Canada (NRKW) to assign demographic variables. These databases contain extensive maternal familial relationships established through field observations and genetic testing (described by Towers et al. 2015). For each adult male (≥12 years), we determined whether his mother was living and, for adult females (≥12 years), whether the individual had a living calf (≤3 years). We identified 3 years as the cutoff age for a calf because it approximated the mean calving interval for adult females during the period in which the tagging was conducted.

### Statistical analyses

We constructed linear and generalized linear mixed effects models (LMM and GLMM) in R v.3.6.3 (R Core Team 2020) using the lme4 (Bates et al. 2015) and glmmTMB (Brooks 2017) packages. We constructed separate models with 1) all tag deployments (models a–f, Table 2) and 2) subsets of deployments to explore demographic effects of calf presence (adult females only, model g, Table 2) and living mother presence (adult males only, model h, Table 2). We constructed individual full models with fixed effects of 1) population, sex, and their interaction (models a–f, Table 2), 2) population, presence of calf, and their interaction (for adult females, model g, Table 2), and 3) population, presence of living mother, and their interaction (for adult males, model h, Table 2), and we included offset effects of the log-transformed deployment duration (models a, g, and h) and the square root-transformed cumulative searching time (model b, Table 2) for those models that contained counts as response variables. For beta regression models we transformed values of 0 and 1 following methods of Duoma and Weedon (2019), and we tested for fixed versus variable dispersion by comparing AIC scores for a model with fixed (null) dispersion to those with population, sex, or population:sex as variable dispersion terms (models c and d, Table 2), and retained the model with the lowest AIC score. For the beta regression model of the proportion of time spent in prey capture dives, the null dispersion model was optimal. For the beta regression model of the proportion of time spent traveling or resting, the model with sex as a dispersion term was optimal. Model response variables included total number of prey capture dives within a deployment (negative binomial and Poisson distributions, models a, b, g, and h, Table 2), proportion of deployment time engaged in prey capture dives (beta distribution, model c, Table 2), proportion of deployment time engaged in traveling or resting dives (beta distribution, model d, Table 2), maximum depth of a prey capture dive (Gaussian distribution, log-transformed to meet model assumptions, model e, Table 2), and bathymetry at the location of a prey capture dive (Gaussian distribution, log-transformed to meet model assumptions, model f, Table 2). For the models with count data as the response variable (models a, b, g, and h, Table 2), we explored several candidate models with Poisson and negative binomial distributions, with and without terms for overdispersion and zero inflation, and used AIC model selection to identify the optimal models. Additionally, we followed the protocol outlined by Zuur et al. (2009) to identify the optimal random structure, which involves including all reasonable random effects that are potentially important, constructing models with different permutations of these random effects, and using AIC model selection to identify the model with the lowest AIC score. There is debate over the appropriate threshold to use in AIC model selection (Grueber et al. 2011; Harrison et al. 2018). Earlier work suggested that small delta thresholds of 2 or less should be used to reduce the likelihood of including overly complex models with unnecessary predictors (Burnham and Anderson 2002), while later work suggested that larger delta thresholds may be necessary in certain cases, especially if models are overdispersed (e.g., Richards 2008; Bolker et al. 2009; Burnham et al. 2011). To identify the optimal random structure, we selected models with the lowest AIC score using a delta threshold of 2, and performed tests using the DHARMa package (Hartig 2020) to verify that our models were not overdispersed. This accepted approach provides a repeatable method by which to systematically eliminate nonsignificant random effects. We considered random effects of age (continuous), deployment year (factor), week-year (factor), and deployment ID (factor), alone and in combination. Deployment ID was defined as the unique tag deployment on an individual, and was used to control for the assumption that dives within deployments should be more similar than dives between deployments. This term allowed us to control for pseudoreplication for all models in which dive was the unit of analysis (models e and f, Table 2). Week-year was defined as the week of the year in which a given tag was deployed. There was no numerical relationship between week-year levels. Rather, this term accounted for the assumption that groups within the same week-year level were more similar than those from different levels, and allowed us to account for unmeasurable environmental variation between populations, especially variation in salmon abundance. It was not appropriate to use a continuous variable that could capture the potential covariance between weeks given that the study period spanned only a few weeks per study site each year. It is important to note that while week-year accounted for temporal nonindependence of observations across the study, deployment ID further accounted for potential spatiotemporal nonindependence within a given week-year since dives within the same deployment should be more similar than those between deployments.

**Table 1:** Summary of analyzed Dtag deployments in Northern and Southern resident killer whale populations between 2009 and 2014, ordered by population, sex, and age

Deployment	Date and time (yyyy-mm-dd hh:mm:ss)	Pop.	Sex	Age	Deployment duration (h)	No. dives analyzed	No. prey cap. dives	Calf^a^ (years)	Mother alive^b^ (years)	Time searching (h)^c^
oo10_260a	2010-09-17 11:25:12	NRKW	F	8	6.50	605	26	—	—	1.05
oo11_240a	2011-08-28 15:28:04	NRKW	F	9	3.77	363	4	—	—	1.24
oo09_235a	2009-08-23 14:17:32	NRKW	F	10	2.74	317	8	—	—	1.83
oo10_256a	2010-09-13 11:03:10	NRKW	F	10	7.13	821	35	—	—	3.40
oo11_224a	2011-08-12 08:51:17	NRKW	F	10	1.92	225	5	—	—	0.38
oo09_237c	2009-08-25 12:35:58	NRKW	F	12	0.97	95	2	1	—	0.09
oo09_247a	2009-09-04 10:27:05	NRKW	F	15	1.05	123	2	0	—	0
oo09_231a	2009-08-19 11:58:16	NRKW	F	16	7.01	539	16	N	—	—
oo10_265a	2010-09-22 15:31:15	NRKW	F	20	2.78	302	21	N	—	2.14
oo11_246a	2011-09-03 12:46:50	NRKW	F	30	3.50	441	18	0	—	2.16
oo11_267a	2011-09-24 11:01:53	NRKW	F	36	6.87	622	12	1	—	—
oo11_248a	2011-09-05 13:10:15	NRKW	M	6	0.35	29	0	—	—	0
oo11_248b	2011-09-05 13:53:25	NRKW	M	7	2.74	306	0	—	—	0.39
oo11_244b	2011-09-01 13:03:11	NRKW	M	11	0.77	69	2	—	—	0
oo09_239a	2009-08-27 11:41:28	NRKW	M	13	2.00	150	2	—	29	0.14
oo12_235b	2012-08-22 14:21:59	NRKW	M	16	4.31	471	11	—	32	2.70
oo09_245b	2009-09-02 17:51:45	NRKW	M	21	1.31	123	5	—	38	0.54
oo09_244a	2009-09-01 15:14:42	NRKW	M	22	3.92	297	19	—	41	1.45
oo09_237d	2009-08-25 16:09:04	NRKW	M	23	3.03	320	9	—	N	2.06
oo09_236a	2009-08-24 15:37:43	NRKW	M	24	2.18	158	1	—	38	0.54
oo09_245a	2009-09-02 13:34:01	NRKW	M	24	5.52	488	16	—	N	2.54
oo10_264a	2010-09-21 17:18:03	NRKW	M	24	1.48	125	2	—	N	0.11
oo11_244a	2011-09-01 09:24:22	NRKW	M	26	2.69	180	0	—	55	0.76
oo09_234a	2009-08-22 15:26:55	NRKW	M	27	3.72	348	7	—	N	2.65
oo11_245a	2011-09-02 07:58:13	NRKW	M	28	11.21	859	10	—	42	2.05
oo09_243a	2009-08-31 16:21:36	NRKW	M	29	2.80	234	7	—	43	0.63
oo11_224b	2011-08-12 16:19:13	NRKW	M	29	0.21	15	1	—	54	0.06
oo09_240a	2009-08-28 11:51:51	NRKW	M	32	3.31	341	13	—	N	2.24
oo10_261a	2010-09-18 15:14:55	NRKW	M	39	2.91	296	5	—	62	2.37
oo09_238a	2009-08-26 07:59:15	NRKW	Unk	3	11.19	1019	13	—	—	3.22
oo14_249m	2014-09-06 09:55:10	SRKW	F	5	5.58	567	10	—	—	1.30
oo12_266m	2012-09-22 10:39:21	SRKW	F	17	2.39	214	10	N	—	1.20
oo10_268m	2010-09-25 10:53:31	SRKW	F	19	7.13	629	3	N	—	—
oo12_267m	2012-09-23 14:56:07	SRKW	F	19	2.29	244	0	3	—	1.11
oo14_264m	2014-09-21 11:31:46	SRKW	F	19	0.65	60	2	N	—	0.45
oo10_264m	2010-09-21 12:37:09	SRKW	F	20	2.45	217	0	3	—	0
oo10_261m	2010-09-18 15:32:45	SRKW	F	24	0.61	36	0	N	—	0.16
oo10_267m	2010-09-24 14:34:45	SRKW	F	36	3.77	278	0	1	—	—
oo12_266n	2012-09-22 13:45:09	SRKW	F	38	0.42	51	0	2	—	0.35
oo12_250m	2012-09-06 10:51:13	SRKW	F	41	6.38	595	18	N	—	—
oo12_260m	2012-09-16 12:24:02	SRKW	M	2	2.67	153	3	—	—	—
oo10_259m	2010-09-16 15:50:54	SRKW	M	6	1.51	196	5	—	—	—
oo10_251m	2010-09-08 14:40:22	SRKW	M	7	0.99	117	3	—	—	0.46
oo10_265m	2010-09-22 12:15:42	SRKW	M	9	6.04	517	25	—	—	2.90
oo12_251m	2012-09-07 11:22:21	SRKW	M	11	1.53	145	8	—	—	0.87
oo14_266m	2014-09-23 10:53:41	SRKW	M	12	4.35	455	13	—	29	3.92
oo12_254m	2012-09-10 10:46:44	SRKW	M	16	6.46	574	9	—	N	3.73
oo10_257m	2010-09-14 14:00:35	SRKW	M	17	4.22	516	24	—	50	—
oo10_270m	2010-09-27 12:47:05	SRKW	M	21	1.01	111	5	—	50	—
oo14_250m	2014-09-07 09:52:25	SRKW	M	21	8.39	822	17	—	43	—
oo12_261m	2012-09-17 10:11:55	SRKW	M	22	2.02	174	3	—	N	0.78
oo14_263m	2014-09-20 11:57:15	SRKW	M	23	6.08	502	11	—	N	2.91

^a^Adult females (≥12 years) with or without (N) a living calf (≤3 years). Estimated age (years) is provided for all calves of tagged adult females.

^b^Adult males (≥12 years) with or without (N) a living mother at time of tagging. Estimated age (years) is provided for living mothers.

^c^Empty cells indicate deployments in which audio recordings could not be analyzed.

**Table 2:** Summary statistics for best-fit models of foraging-related response variables for Northern and Southern Resident killer whales 2009–2014

Model ID	Response	Model type	Model family	Random effects	Offset effects	Dispersion parameter	Fixed effects	Obs. unit	*n*	df	Estimate	s.e.	*t* value or *z* value	*P* value
a	No. prey capture dives	GLMM	Negative binomial (nbinom1)	Week of year	log(deployment duration)	—	Population	Deployment	51	49	−1.1086−0.5990	0.3559	−3.115	0.0018
							Sex					0.2555	−2.344	0.0191
							Population:sex				1.5055	0.4358	3.454	0.0006
b	No. prey capture dives	GLMM	Negative binomial (nbinom1)	Week of year	sqrt(cumulative searching time)	—	Population	Deployment	41	39	−1.4160−0.7635	0.4996	−2.834	0.0046
							Sex					0.2664	−2.866	0.0042
							Population:sex				1.6041	0.5788	2.771	0.0056
c	Prop. time engaged in prey capture	GLMM	Beta	Week of year	—	—	Population	Deployment	51	49	−1.045	0.383	−2.73	0.0063
							Sex				−0.379	0.296	−1.28	0.2001
							Population:sex				1.471	0.470	3.13	0.0017
d	Prop. time engaged in travel or rest	GLMM	Beta	Week of year	—	sex	Population	Deployment	51	48	−1.035	0.375	−2.76	0.0058
							Sex				−0.356	0.324	−1.10	0.2722
											0.715	0.354	2.02	0.043
e	log(max. depth)	LME	Gaussian	Week of year	—	—	Population	Dive	428	40	0.4531	0.2066	2.1934	0.0342
				Deployment			Sex			40	0.0390	0.2075	0.1879	0.8519
f	log(bathym)	LME	Gaussian	Week of year	—	—	Population	Dive	414	38	−0.2187	0.1499	−1.4591	0.1528
				Deployment			Sex			38	0.3542	0.1482	2.3903	0.0219
g	No. prey capture dives by adult females	GLMM	Poisson	Week of year	log(deployment duration)	—	Population	Deployment	15	14	−2.1826	0.7091	−3.0780	0.0021
							Calf			14	−1.9471	0.7175	−2.7140	0.0067
h	No. prey capture dives by adult males	GLMM	Poisson	Week of year	log(deployment duration)	—	Population	Deployment	22	21	−0.2347	0.4060	−0.5780	0.5632
							Living mother			21	−0.1769	0.2175	−0.8140	0.4160
							Population:living mother			21	0.8554	0.3946	2.1680	0.0302

For the model examining the relationship between the presence of a living mother and the number of prey capture dives by adult males (model h, Table 2), we additionally explored the importance of including a random effect of the categorical age of the tagged male’s mother (dead, reproductive, or post-reproductive), but there was no support to include this random effect in the final model. The best models included week-year (all models, Table 2) and deployment ID (models e and f, Table 2) as random effects. We used recursive, single term deletion and model comparison of successively simpler models using Likelihood Ratio Tests to determine which fixed effects to omit from the final models. We used Tukey HSD tests to compare levels of model effects (see Supplementary Information, Table S2). For all tests, α = 0.05. Where relevant in statistical analyses, we omitted one NRKW deployment (oo09_238a) for which sex was unknown because this juvenile died before its sex could be determined.

We were unable to use available salmon abundance indices due to 1) spatial incongruency between the salmon stocks/regions where these abundance data were sampled and the prey consumed by NRKW and SRKW during our study period and location, 2) issues with sporadic coverage and the degree to which effort had been accounted for in some of the existing salmon indices, and 3) reliability issues with applying widely used salmon data from the Albion Test Fishery situated on the Fraser River (e.g., Ayres et al. 2012; Ford et al. 2016; Wasser et al. 2017; Holt et al. 2021a), because of uncertainty in the proportions of salmon taking one of two possible paths around Vancouver Island, BC to return to the Fraser River (Figure 1). Therefore, the week-year random effect allowed us to account as best as possible for temporal variability in salmon abundance and other environmental factors on a weekly basis that might be responsible for the differences in foraging patterns we measured within and between populations. However, we cannot rule out the possibility that longer-term differences in salmon abundance and availability between populations could have driven the observed differences in foraging strategies.

## RESULTS

We analyzed 186.8 h of dive data from 52 Dtag deployments (109.9 h from 30 NRKW, 76.9 h from 22 SRKW; Figure 1). Mean deployment durations for NRKW and SRKW were 3.7 h (range = 0.2–11.2 h) and 3.5 h (range = 0.4–8.4 h), respectively. Of the total deployments, 30 were on males (54.4 h on 18 NRKW, 45.2 h on 12 SRKW), 21 were on females (44.3 h on 11 NRKW, 31.7 h on 10 SRKW), and one was on an individual of unidentified sex (11.2 h on NRKW; Table 1).

### Foraging ecology

**Figure 2:**
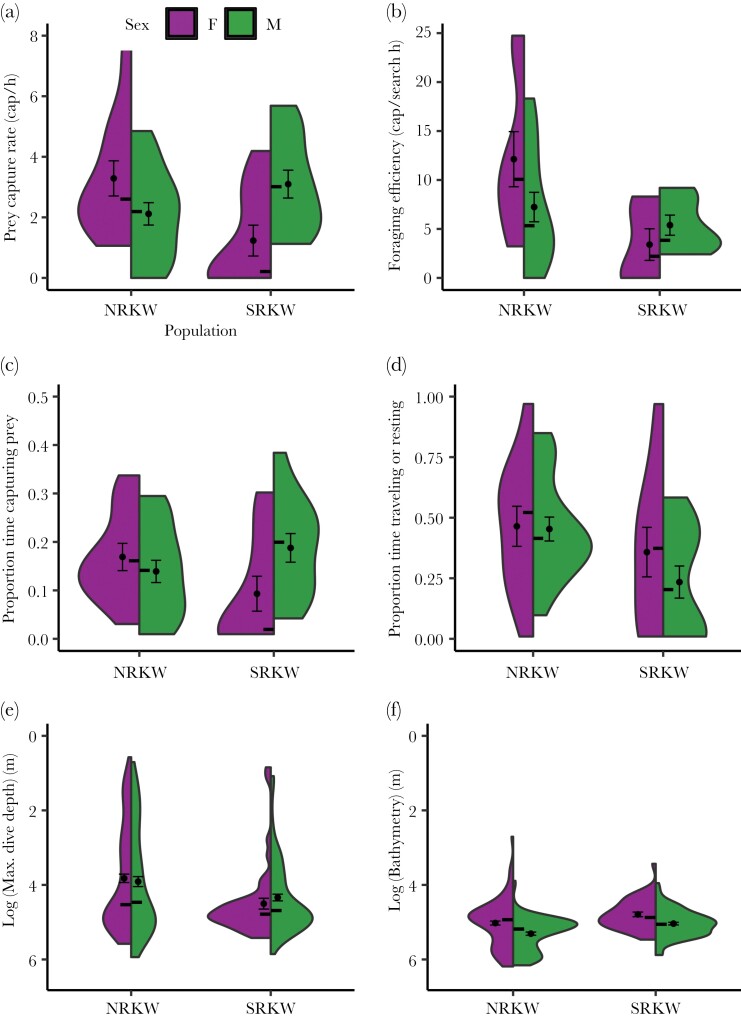
The relationship between sex and foraging behavior differed between killer whale populations. Violin plots depict trimmed kernel densities, points and bars depict means ± standard errors, and horizontal lines indicate median values for (a) total prey capture dives standardized by deployment duration in hours (NRKW female: *n* = 11; NRKW male: *n* = 18; SRKW female: *n* = 10; SRKW male: *n* = 12), (b) total prey capture dives standardized by searching effort in hours (NRKW female: *n* = 8; NRKW male: *n* = 16; SRKW female: *n* = 6; SRKW male: *n* = 7), (c) proportion of deployment time spent capturing prey (NRKW female: *n* = 11; NRKW male: *n* = 18; SRKW female: *n* = 10; SRKW male: *n* = 12), (d) proportion of deployment time spent traveling or resting (NRKW female: *n* = 11; NRKW male: *n* = 18; SRKW female: *n* = 10; SRKW male: *n* = 12), (e) log of the maximum depth of prey capture dives (m; NRKW female: *n* = 149; NRKW male: *n* = 110; SRKW female: *n* = 43; SRKW male: *n* = 126), and (f) log of the bathymetry at the location of prey capture dives (m; NRKW female: *n* = 149; NRKW male: *n* = 110; SRKW female: *n* = 42; SRKW male: *n* = 113).

There was a significant interaction between population and sex on the number of prey capture dives, offsetting for deployment duration (GLMM, *z* = 3.454, *P* = 0.0006, Table 2, Supplementary Table S2, presented in Figure 2a as prey capture rate, prey capture dives per hour). NRKW females captured 167% more prey per hour than SRKW females. Additionally, SRKW males captured 152% more prey per hour than SRKW females, while there was an opposite trend that NRKW females captured 55% more prey per hour than NRKW males (observed mean values of prey capture dives per h, NRKW F: 3.29, NRKW M: 2.12, SRKW F: 1.23, SRKW M: 3.10). These trends were not due to differences in effort. Indeed, when controlling for cumulative time spent searching for prey there was a significant interaction between population and sex on the number of prey capture dives (GLMM, *z* = 2.771, *P* = 0.0056, Table 2, Supplementary Table S2, presented in Figure 2b as foraging efficiency, prey capture dives per h searching). NRKW females were 257% more efficient than SRKW females and 68% more efficient than NRKW males, while there was an opposite trend that SRKW males were 59% more efficient than SRKW females (observed mean values of prey capture dives per h searching, NRKW F: 12.13, NRKW M: 7.24, SRKW F: 3.40, SRKW M: 5.39).

Time spent engaged in prey capture versus traveling and resting dives differed between populations. There was an interaction between population and sex on the proportion of deployment time engaged in dives that resulted in prey capture (GLMM, *z* = 3.13, *P* = 0.0017, Table 2, Supplementary Table S2). NRKW females spent 91% and 23% more time engaged in prey capture dives than SRKW females or NRKW males, respectively. In contrast, SRKW males spent 114% more time engaged in prey capture dives than SRKW females (Figure 2c) (observed mean values of proportion of deployment spent in prey capture dives, NRKW F: 0.162, NRKW M: 0.132, SRKW F: 0.085, SRKW M: 0.182). Additionally, there was a population effect on the proportion of tag deployment time that a subject spent traveling or resting (GLMM, *z* = −2.76, *P* = 0.0058, Table 2, Supplementary Table S2). Across both sexes, NRKW engaged in traveling or resting behavior for 62% more time than SRKW (Figure 2d) (observed mean values of proportion of deployment spent in travel or resting dives, NRKW: 0.463, SRKW: 0.286).

Dive depth was an important factor contributing to population differences in foraging behavior. There was a significant effect of population on the log of the maximum depth of prey capture dives (LMM, *t* = 2.1934, *P* = 0.0342, Table 2, Supplementary Table S2). Average depth of SRKW prey capture was 20% greater than NRKW prey capture depth (Figure 2e) (observed mean values of maximum depth of prey capture dives, NRKW: 90.63, SRKW: 108.54). This difference was not explained by bathymetry, as the foraging habitats used by the two populations did not differ in depth. Additionally, there was an effect of sex on the log of the foraging habitat depth (LMM, *t* = 2.3903, *P* = 0.0219, Table 2, Supplementary Table S2). Across both populations, males tended to make prey capture dives in areas that were 13% deeper than areas in which females foraged (Figure 2f) (observed mean values of habitat depth at location of prey capture dive, F: 171.36, M: 193.12).

### Demographic effects on foraging ecology

**Figure 3:**
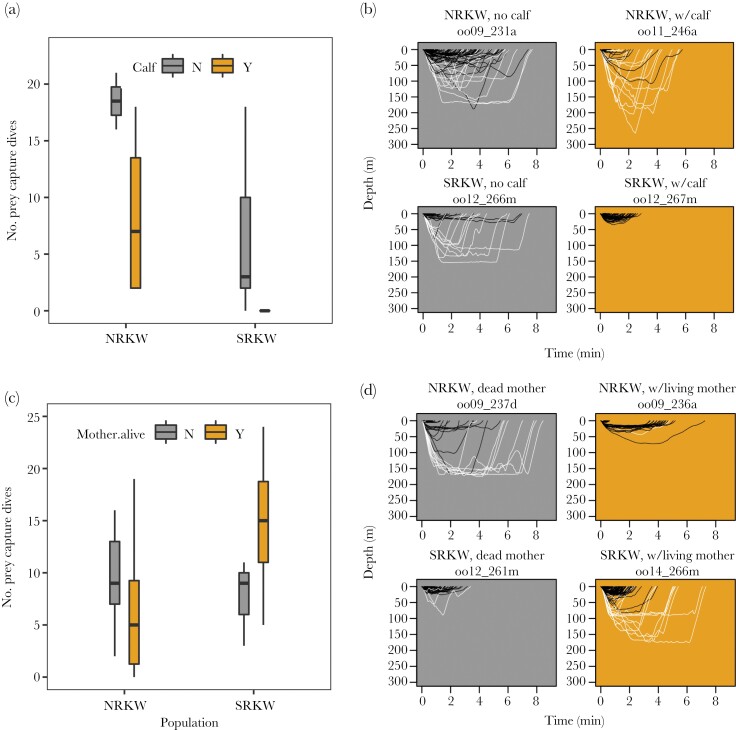
Demography affected the number of prey capture dives. (a, b) In both killer whale populations, the presence of a calf reduced the number of prey captured by adult females, and the effect was greatest for SRKW (NRKW no calf: *n* = 2; NRKW w/calf: *n* = 4; SRKW no calf: *n* = 5; SRKW w/calf: *n* = 4). (c, d) For NRKW, adult males with a living mother captured fewer prey, whereas for SRKW males, those with a living mother captured more prey (NRKW dead mother: *n* = 5; NRKW alive mother: *n* = 10; SRKW dead mother: *n* = 3; SRKW alive mother: *n* = 4). Boxplots (a, c) display median (horizontal line), interquartile range (boxes), and observations within 1.5 times the interquartile range (whiskers). Dive plots (b, d) depict all dives (white = prey capture, black = other) from representative deployments on tagged whales (gray shading = absence [of calf or living mother], gold shading = presence).

Population and the presence of a calf were significant predictors of the number of prey capture dives by adult females (GLMM, population: *z* = −3.078, *P* = 0.0021; calf: *z* = −2.714, *P* = 0.0067, Table 2, Supplementary Table S2). Across both populations, females without calves made more prey capture dives than females with calves. Accounting for deployment duration, NRKW adult females without calves made 133% more prey capture dives per hour than SRKW adult females without calves and 81% more than NRKW females with calves. NRKW females with calves captured more prey than SRKW females with calves, who captured no prey while tags were attached (Figure 3a,b) (observed mean values of prey capture dives per h, NRKW no calf: 4.91, NRKW with calf: 2.71, SRKW no calf: 2.11, SRKW with calf: 0). There was a significant interaction between population and the presence of a living mother on the number of prey capture dives by adult males (GLMM, *z* = 2.168, *P* = 0.0302, Table 2, Supplementary Table S2). Accounting for deployment duration, SRKW males with a living mother made 151% more prey capture dives per hour than SRKW males whose mother had died, whereas the opposite was true for NRKW, whereby NRKW whose mother had died made 17% more prey capture dives per hour than NRKW males with a living mother (Figure 3c,d) (observed mean values of prey capture dives per h, NRKW dead mother: 2.61, NRKW living mother: 2.24, SRKW dead mother: 1.56, SRKW living mother: 3.92).

## DISCUSSION

Empirical studies investigating the partitioning of individual responsibilities within and between groups can advance an understanding of the factors promoting and maintaining diversity in behavior, and can inform predictions about how novel pressures may impact wildlife. Here, we demonstrate that patterns of individual foraging behavior by resident killer whales are not fixed across populations. We use kinematic and acoustic data from high-resolution bio-logging tags to reveal striking population differences in the sex-based and demographic patterns of individual foraging behavior. We demonstrate that the NRKW population, which has grown nearly continuously for the past two decades, employed a female foraging strategy characterized by females exhibiting a greater number of prey capture dives, greater foraging efficiency, and a larger proportion of time spent capturing prey compared with males, consistent with previous work (Wright et al. 2016). In contrast, in the SRKW population, which to date has exhibited virtually no net growth since annual censusing efforts began in 1976, female foraging behavior was greatly reduced, especially for females with dependent young. Moreover, there was a trend of greater prey capture by adult males, especially those with a living mother, and SRKW spent less time resting or traveling compared with NRKW.

The interpopulation differences in individual foraging behavior could be driven by fluctuations in population size and the concomitant changes in social dynamics. SRKW have experienced high mortality, including the loss of critical information-bearing post-reproductive matriarchs. In gregarious species, older group members often serve as “keystone individuals” that exert disproportionate influence on group behavior and stability of social hierarchies (McComb et al. 2001; Modlmeier et al. 2015; Wooddell et al. 2016; Busson et al. 2019). Given that the role of post-reproductive matriarchs as leaders is likely critical in resident killer whale populations, the chance loss of key individuals and their personalities (i.e., repeated behavioral syndromes; Sih et al. 2004) could affect patterns and outcomes of behavior (Keiser and Pruitt 2014).

Alternatively, differences in individual foraging roles across populations may be the outcome of environmental pressures differentially impacting population growth rates, which may in turn affect behavioral strategies. SRKWs have experienced high levels of anthropogenic disturbance (NMFS 2016) including the disproportionate impact of vessel presence on female foraging compared with male foraging (Holt et al. 2021b). These differences in disturbance have been implicated in the divergent population growth trajectories (Murray et al. 2021). Consequently, the decomposition of robust social structure within the SRKW (Williams and Lusseau 2006; Busson et al. 2019), potentially mediated by greater disturbance, could have shifted the cost–benefit tradeoffs underlying foraging strategies.

We demonstrate an effect of calf presence on prey capture across both populations. Adult females with a calf captured prey less than those without, and the effect of calf presence on foraging was more pronounced in SRKW. None of the SRKW mothers with calves engaged in any prey capture attempts at depth during the study period, whereas all NRKW mothers with calves continued to make prey capture dives, albeit fewer than the NRKW females without calves. Foragers must routinely balance the competing strategies of either conserving energy stores to minimize the likelihood of starvation (robust satisficing) or maximizing energy obtained from foraging (optimizing) (Carmel and Ben-Haim 2005). For SRKW experiencing scarce and patchy resources and uncertainty in prey capture due to the depletion of many Pacific salmon stocks (Brown et al. 2019; Hanson et al. 2021), robust satisficing by mothers with calves (conserving energy by conducting prey capture dives less frequently and potentially receiving prey from other individuals more often) may be favored (Carmel and Ben-Haim 2005). Additional data are needed to tease out the nuances of the effect of calf presence, given the relatively small sample sizes of deployments on females with calves. Furthermore, we cannot rule out the possibility that the presence of the tagging vessel influenced foraging behavior, especially of mothers with depending young. However, once the tag was applied, the tagging vessel typically remained at distances comparable to other commercial whale watching vessels in the area. Therefore, any impact from the tagging vessel may underscore the general sensitivity of lactating females to nearby vessels (e.g., Holt et al. 2021b). Given the number of vessels frequently in their proximity (Giles 2014; Holt et al. 2021a, 2021b), SRKW females with vulnerable calves may have been routinely foregoing foraging opportunities during the study period. Whether this loss of opportunity to consume prey is offset by food sharing from other individuals in the pod or translates to energy loss remains unknown.

The finding that adult SRKW males captured more prey if their mother was alive highlights the opposite patterns of foraging behavior we observed for adult males between the two populations. Males require more energy due to their larger size (Noren 2011), and the tendency for NRKW mothers to provision their adult male offspring should maximize maternal inclusive fitness (Wright et al. 2016), since older males sire a disproportionate number of offspring in resident killer whale populations (Ford et al. 2011). In the absence of living mothers, NRKW adult males may have to forage more to make up for the lack of maternal provisioning, which aligns with our findings for this population. In contrast, SRKW adult males with a living mother captured more prey than SRKW adult males whose mothers had died and NRKW adult males with a living mother, while there was no apparent difference in the number of prey captured by NRKW and SRKW males whose mothers had died. It is unlikely that the increased prey capture by SRKW males served the purpose of sharing prey to offset the lactation costs of their mothers, since none of the mothers of tagged SRKW adult males had calves (≤3 years), and the majority were post-reproductive (≥40 years). Instead, it is possible that greater prey capture by SRKW adult males with living mothers may be a strategy to help offset their post-reproductive mother’s reduced foraging effort which we documented in SRKW females. Given the benefits of post-reproductive matriarch survival (McComb et al. 2001; Modlmeier et al. 2015; Wooddell et al. 2016; Busson et al. 2019; Nattrass et al. 2019), it is possible that adult sons in demographically unbalanced, endangered populations may attempt to promote survival of matrilineal members including their mothers, for example through prey-sharing. This hypothesis is supported by the fact that survival of male resident killer whales is impacted by maternal death (Foster et al. 2012). Moreover, prey-sharing between individuals is common in resident killer whales (Wright et al. 2016), and prey-sharing by adult males with their mothers and siblings has been observed in NRKW presumably as a form of pseudoreciprocity, albeit much less often than females provisioning related males (Wright et al. 2016). It is therefore possible that SRKW adult males engage in capturing prey and potentially sharing with maternally related group members including their mothers, while SRKW adult females engage in context-dependent robust satisficing to reduce overall energy use, particularly in areas of intensified anthropogenic pressure. This potential prey-sharing role of SRKW adult males challenges the existing paradigm that adult females are disproportionate provisioners in resident killer whale populations. However, if adult males are provisioning their mothers, one might expect increased survival probability of sons with the death of their mothers due to the released foraging burden. Rather, we see the opposite—that male survival probability decreases with maternal death (Foster et al. 2012). Additional evidence would be helpful for testing the hypothesis that sons share prey with their mothers, such as 1) demonstrated prey-sharing by SRKW adult males, either directly via observations (e.g., as in NRKW, Wright et al. 2016) or indirectly through increases in post-reproductive female longevity with the presence of adult sons, and 2) demonstrated increases in indirect benefits to adult males from prey-sharing with maternally related group members.

It is worth considering an alternative, nonmutually exclusive hypothesis related to the role of group leadership by post-reproductive females to explain the increased prey capture by SRKW males with living mothers. Given that post-reproductive females typically lead groups (Brent et al. 2015) and that pod members frequently share prey (Wright et al. 2016), leading the group to locations where adult sons and other related males can maximize prey capture and caloric intake could translate into increased survival of these males, which could maximize female inclusive fitness given that older males sire more calves (Ford et al. 2011). Moreover, we showed that males of both populations foraged in areas with deeper bathymetry than females, consistent with previous work (Beerman et al. 2016), and we revealed that SRKW captured prey at greater depths than NRKW, an effect not due to differences in the bathymetry of their foraging habitats. There may be a potential benefit to deeper diving, given that larger Chinook salmon are typically found at greater depths than smaller, less lipid-rich salmonids (Wright et al. 2017). SRKW adult males who routinely forage in deeper areas where searching for large Chinook is more likely to pay off may have greater access to larger prey of higher caloric value than SRKW females who tend to forage in shallower areas to remain with dependent calves and tend to be more sensitive to nearby vessels (Holt et al. 2021b). Consequently, females may lead the group to areas where their sons may have the greatest opportunity to feed on deeper, higher-quality prey, at the cost of reduced foraging by other pod members in vessel-disturbed areas.

Differences in prey abundance and availability between populations could have contributed to the differences in foraging strategies we document here. However, we could not use existing indices of salmon abundance in our analyses because these metrics do not address spatiotemporal uncertainty in the distributions of Chinook salmon returning along one of two paths to the Fraser River. The nuances of migration paths taken are directly related to accurately estimating salmon availability for each population, yet data at this fine-scale spatiotemporal resolution are not currently available. We suggest that future research should elucidate the complex relationships between 1) environmental factors including location, prey distributions, and availability, 2) the behavioral context of the animal at the time of tagging, and 3) differential population-level impacts of anthropogenic pressures, including impacts from tagging and vessel presence, on foraging behavior (e.g., Holt et al. 2021a, 2021b). Furthermore, it is important to determine whether population differences in individual foraging behavior persist throughout space and time, and whether individual roles are flexible within populations.

This study revealed considerable differences in sex-based and demographic patterns of individual foraging strategies, demonstrating that these strategies are not fixed across two ecologically similar populations of a gregarious marine predator. The total number of prey capture dives and proportion of time capturing prey was female biased in the growing NRKW population, and male biased in the endangered SRKW population. The presence of a calf reduced female foraging activity across populations, but disproportionately so for SRKW females. Adult SRKW males with a living mother exhibited increased foraging behavior, possibly as a compensation strategy to offset their pod’s caloric deficits and/or as an outcome of matriarch-led navigation to areas that promote greater prey capture by their adult sons. Interpopulation differences in individual roles within social groups may have arisen with the loss of key individuals as populations shrink, or may provide small, unbalanced populations experiencing environmental stressors the necessary flexibility to respond to novel and unpredictable environments caused by human-induced rapid environmental change. Indeed, interpopulation variation in individual roles may be an important evolutionary strategy for promoting resilience in the face of change.

## Supplementary Material

arad002_suppl_Supplementary_Material

## Data Availability

Analyses reported in this article can be reproduced using the data provided by Tennessen et al. (2022).
