# Supplementary material for: Divergent foraging strategies between populations of sympatric matrilineal killer whales
Source: Behav Ecol. 2023 Mar 4;34(3):373–86. doi: 10.1093/beheco/arad002 (PMC10183210; doi:10.1093/beheco/arad002)
Supplement: arad002_suppl_Supplementary_Material [file arad002_suppl_supplementary_material.docx]

**SUPPLEMENTARY INFORMATION**

**Methods**

Prey capture detection

We identified prey capture dives using detection filters customized separately for each population, with equal sensitivity between populations. Filters removed all dives that did not meet minimum thresholds for three variables that were previously demonstrated to be important predictors of prey capture: jerk peak (maximum peak of the jerk signal - rate of change of acceleration - adjusted by the median jerk signal; Tennessen et al. 2019, Ydesen et al. 2014, Allen et al. 2016, Arranz et al. 2016), roll at jerk peak (absolute value of the animal’s roll angle at the time of jerk peak, in degrees), and circular variance in the animal’s heading during the bottom phase of a dive , computed using the ‘circ_var’ function in the CircStat package (Berens 2009) in MATLAB. We specified the filter thresholds for each variable based on the known prey capture events verified by the acoustic data. Previous research demonstrated that acoustic data from the Dtags can indicate when prey capture has occurred. As resident killer whales and other toothed cetaceans close in on their salmonid prey, they emit buzzes (inter-click interval ≤ 10 ms), associated with the final pursuit phase of foraging. Once prey have been captured, resident killer whales often generate tearing and crunching sounds associated with prey handling as they bring it to the surface to share (Holt et al. 2019). The co-occurrence of buzzes and prey handling sounds is closely related to observations of fish kills at the surface, and has therefore been used to validate prey capture events determined from movement data alone (Tennessen et al. 2019). Due to masking from underwater vessel noise or sounds of water flowing past the tag, acoustic data are not available for all dives in all deployments whereas movement data are robust to these noise sources and provide a more complete record across all deployments. We therefore used the acoustic data to parameterize the filters through which we ran the movement data. We used the subset of dives containing the co-occurrence of buzzes and prey handling sounds to define thresholds for jerk peak, roll and heading variance, specific to each population.

Fundamental to the aim of our study was the need to maximize confidence in our ability to detect prey capture events. Therefore, we sought to maximize our hit rate (true positive rate) and minimize our miss rate (false negative rate). To do this, we defined the filter thresholds for jerk peak, roll and heading variance as the minimum values of each of these variables from the subset of acoustically-confirmed prey capture dives described above (data presented in Table S1), respective to each population (NRKW: n=59, jerk peak = 15.495, roll = 2.61°, heading = 2.58°; SRKW: n = 45, jerk peak = 8.598, roll = 13.55°, heading variance = 22.44°). We omitted two outlier dives from the SRKW acoustic data (one from deployment oo12_266m and one from deployment oo14_266m) in which the kinematic behavior was not consistent with known prey capture movement (Wright et al. 2017, Tennessen et al. 2019), and both dives occurred near the surface. This suggests that the buzzes and prey handling sounds that occurred in these dives could have been from a nearby individual and not the tagged whale, or could have been the result of a fish that had briefly escaped and was then recaptured by the tagged whale or a nearby conspecific rather than initial prey capture by the tagged whale. Given the ambiguity, we could not include these observations in the acoustic data. We computed true positive and false positive rates for each population, by comparing the filtered prey capture dives against the set of acoustically-confirmed prey capture dives. Using this approach, we achieved true positive rates of 100% for both NRKW and SRKW, and false positive rates of 26.4% and 25.7% for NRKW and SRKW, respectively, for dives ≥ 50 m.

**SUPPLEMENTARY REFERENCES**

Allen AN, Goldbogen JA, Friedlaender AS, Calambokidis J. 2016. Development of an automated method of detecting stereotyped feeding events in multisensor data from tagged rorqual whales. Ecol. Evol. 6:7522-7535.

Arranz P, DeRuiter SL, Stimpert AK, Neves S, Friedlaender AS, Goldbogen JA, Visser F, Calambokidis J, Southall BL, Tyack PL. 2016. Discrimination of fast click-series produced by tagged Risso's dolphins (*Grampus griseus*) for echolocation or communication. J. Exp. Biol. 219:2898-2907.

Berens P, 2009. CircStat: a MATLAB toolbox for circular statistics. J. Stat. Softw. 31:1-21.

Holt MM, Hanson MB, Emmons CK, Haas DK, Giles DA, Hogan JT. 2019. Hogan. Sounds associated with foraging and prey capture in individual fish-eating killer whales, *Orcinus orca*. J. Acoust. Soc. Am. 146:3475-3486.

Tennessen JB, Holt MM, Hanson MB, Emmons CK, Giles DA, Hogan JT. 2019. Kinematic signatures of prey capture from archival tags reveal sex differences in killer whale foraging activity. J. Exp. Biol. 222:jeb191874.

Wright BM, Ford JK, Ellis GM, Deecke VB, Shapiro AD, Battaile BC, Trites AW. 2017. Fine-scale foraging movements by fish-eating killer whales (*Orcinus orca*) relate to the vertical distributions and escape responses of salmonid prey (*Oncorhynchus* spp.). Mov. Ecol. 5:3.

Ydesen KS, Wisniewska DM, Hansen JD, Beedholm K, Johnson M, Madsen PT. 2014. What a jerk: prey engulfment revealed by high-rate, super-cranial accelerometry on a harbour seal (*Phoca* vitulina). J. Exp. Biol. 217:2239-2243.

**SUPPLEMENTARY TABLES**

Table S1. Metadata and prey capture detection variables from acoustically confirmed prey capture dives of foraging killer whales.

| **Deployment** | **Population** | **Whale ID** | **Sex** | **Age** | **Max. depth**  **(m)** | **Dive duration**  **(min)** | **Jerk peak** | **Roll (deg)** | **Heading variance (deg)** |
| --- | --- | --- | --- | --- | --- | --- | --- | --- | --- |
| oo09_235a | NRKW | A72 | F | 10 | 111.99 | 2.62 | 26.19 | 1.96 | 0.48 |
| oo09_237d | NRKW | I53 | M | 23 | 154.38 | 6.02 | 24.62 | 2.08 | 0.12 |
| oo09_237d | NRKW | I53 | M | 23 | 162.55 | 7.03 | 70.37 | 1.17 | 0.61 |
| oo09_237d | NRKW | I53 | M | 23 | 170.47 | 8.45 | 39.84 | 3.04 | 0.84 |
| oo09_237d | NRKW | I53 | M | 23 | 164.46 | 7.97 | 50.89 | 3.04 | 0.81 |
| oo09_237d | NRKW | I53 | M | 23 | 174.97 | 5.76 | 44.44 | 1.51 | 0.83 |
| oo09_237d | NRKW | I53 | M | 23 | 169.68 | 3.82 | 53.55 | 0.34 | 0.90 |
| oo09_238a | NRKW | I111 | unk | 3 | 155.80 | 4.64 | 15.90 | 0.11 | 0.21 |
| oo09_243a | NRKW | I39 | M | 29 | 317.33 | 5.24 | 42.91 | 0.84 | 0.74 |
| oo09_245a | NRKW | I46 | M | 24 | 353.72 | 6.15 | 44.76 | 0.10 | 0.27 |
| oo09_245a | NRKW | I46 | M | 24 | 141.74 | 3.13 | 58.47 | 0.06 | 0.26 |
| oo09_245a | NRKW | I46 | M | 24 | 111.60 | 3.01 | 70.73 | 0.05 | 0.40 |
| oo09_245a | NRKW | I46 | M | 24 | 124.43 | 3.30 | 44.52 | 0.35 | 0.87 |
| oo09_245a | NRKW | I46 | M | 24 | 161.10 | 3.87 | 52.75 | 0.73 | 0.65 |
| oo09_245a | NRKW | I46 | M | 24 | 154.23 | 4.57 | 47.46 | 0.76 | 0.22 |
| oo09_245a | NRKW | I46 | M | 24 | 300.96 | 6.02 | 39.19 | 1.54 | 0.34 |
| oo09_245a | NRKW | I46 | M | 24 | 116.47 | 2.70 | 23.44 | 0.43 | 0.29 |
| oo09_245b | NRKW | I62 | M | 21 | 93.11 | 3.03 | 25.29 | 1.02 | 0.70 |
| oo10_251m | SRKW | J39 | M | 7 | 128.78 | 3.25 | 35.55 | 1.19 | 0.58 |
| oo10_251m | SRKW | J39 | M | 7 | 116.34 | 3.95 | 48.63 | 2.45 | 0.72 |
| oo10_251m | SRKW | J39 | M | 7 | 165.26 | 4.46 | 34.43 | 0.43 | 0.39 |
| oo10_256a | NRKW | G64 | F | 10 | 174.80 | 4.74 | 39.99 | 2.39 | 0.81 |
| oo10_256a | NRKW | G64 | F | 10 | 144.43 | 4.14 | 64.38 | 3.10 | 0.47 |
| oo10_256a | NRKW | G64 | F | 10 | 161.58 | 4.43 | 19.39 | 2.91 | 0.74 |
| oo10_256a | NRKW | G64 | F | 10 | 238.58 | 4.70 | 24.56 | 0.14 | 0.55 |
| oo10_256a | NRKW | G64 | F | 10 | 146.36 | 4.49 | 42.14 | 2.40 | 0.46 |
| oo10_256a | NRKW | G64 | F | 10 | 152.25 | 4.41 | 41.57 | 2.84 | 0.73 |
| oo10_256a | NRKW | G64 | F | 10 | 149.28 | 4.21 | 30.36 | 2.88 | 0.49 |
| oo10_256a | NRKW | G64 | F | 10 | 118.86 | 3.26 | 29.13 | 2.96 | 0.76 |
| oo10_256a | NRKW | G64 | F | 10 | 171.79 | 3.65 | 32.38 | 0.95 | 0.77 |
| oo10_256a | NRKW | G64 | F | 10 | 134.75 | 5.93 | 41.37 | 1.28 | 0.81 |
| oo10_256a | NRKW | G64 | F | 10 | 141.67 | 7.21 | 41.35 | 2.41 | 0.85 |
| oo10_256a | NRKW | G64 | F | 10 | 105.94 | 4.83 | 48.18 | 2.34 | 0.35 |
| oo10_256a | NRKW | G64 | F | 10 | 112.11 | 3.34 | 25.39 | 2.92 | 0.66 |
| oo10_256a | NRKW | G64 | F | 10 | 113.46 | 4.59 | 21.78 | 0.09 | 0.24 |
| oo10_260a | NRKW | A75 | F | 8 | 137.03 | 3.98 | 76.84 | 1.69 | 0.05 |
| oo10_260a | NRKW | A75 | F | 8 | 120.68 | 3.47 | 89.50 | 2.69 | 0.90 |
| oo10_260a | NRKW | A75 | F | 8 | 123.89 | 2.92 | 29.31 | 1.25 | 0.73 |
| oo10_261a | NRKW | A38 | M | 39 | 152.43 | 2.74 | 84.28 | 0.86 | 0.30 |
| oo10_261a | NRKW | A38 | M | 39 | 176.46 | 6.65 | 116.93 | 1.02 | 0.41 |
| oo10_261a | NRKW | A38 | M | 39 | 159.54 | 3.79 | 40.15 | 1.31 | 0.79 |
| oo10_261a | NRKW | A38 | M | 39 | 177.93 | 3.62 | 107.07 | 2.62 | 0.63 |
| oo10_265a | NRKW | G49 | F | 20 | 130.84 | 3.40 | 26.47 | 2.09 | 0.63 |
| oo10_265m | SRKW | K33 | M | 9 | 186.08 | 3.05 | 45.01 | 1.14 | 0.49 |
| oo10_265m | SRKW | K33 | M | 9 | 112.88 | 4.79 | 48.54 | 3.00 | 0.53 |
| oo10_265m | SRKW | K33 | M | 9 | 119.34 | 3.50 | 50.43 | 0.79 | 0.75 |
| oo10_265m | SRKW | K33 | M | 9 | 100.84 | 6.02 | 40.93 | 2.02 | 0.71 |
| oo10_265m | SRKW | K33 | M | 9 | 130.31 | 4.32 | 61.88 | 2.83 | 0.69 |
| oo10_265m | SRKW | K33 | M | 9 | 122.87 | 3.98 | 78.54 | 1.73 | 0.76 |
| oo10_265m | SRKW | K33 | M | 9 | 117.03 | 3.39 | 47.07 | 1.22 | 0.65 |
| oo10_265m | SRKW | K33 | M | 9 | 136.76 | 3.56 | 49.83 | 2.01 | 0.87 |
| oo10_265m | SRKW | K33 | M | 9 | 155.30 | 4.73 | 68.99 | 1.56 | 0.75 |
| oo10_265m | SRKW | K33 | M | 9 | 47.00 | 1.68 | 14.38 | 2.97 | 0.40 |
| oo11_240a | NRKW | I104 | F | 9 | 200.16 | 4.20 | 54.71 | 2.36 | 0.63 |
| oo11_245a | NRKW | I43 | M | 28 | 164.25 | 4.20 | 79.05 | 1.34 | 0.73 |
| oo11_245a | NRKW | I43 | M | 28 | 46.60 | 2.25 | 17.84 | 0.29 | 0.48 |
| oo11_245a | NRKW | I43 | M | 28 | 190.25 | 4.15 | 36.40 | 0.32 | 0.27 |
| oo11_246a | NRKW | G31 | F | 30 | 202.14 | 5.05 | 110.91 | 1.78 | 0.54 |
| oo11_246a | NRKW | G31 | F | 30 | 121.86 | 2.37 | 49.39 | 3.03 | 0.38 |
| oo11_246a | NRKW | G31 | F | 30 | 265.11 | 4.35 | 48.32 | 0.40 | 0.85 |
| oo11_246a | NRKW | G31 | F | 30 | 131.40 | 5.52 | 21.81 | 2.68 | 0.88 |
| oo11_246a | NRKW | G31 | F | 30 | 204.84 | 3.68 | 69.12 | 2.41 | 0.50 |
| oo11_246a | NRKW | G31 | F | 30 | 181.13 | 3.70 | 24.63 | 2.09 | 0.74 |
| oo11_246a | NRKW | G31 | F | 30 | 166.86 | 2.76 | 15.49 | 1.14 | 0.38 |
| oo11_267a | NRKW | A34 | F | 36 | 138.69 | 4.35 | 20.08 | 2.32 | 0.68 |
| oo12_235b | NRKW | A66 | M | 16 | 104.12 | 6.11 | 62.41 | 0.12 | 0.87 |
| oo12_235b | NRKW | A66 | M | 16 | 135.82 | 3.38 | 35.83 | 0.77 | 0.83 |
| oo12_235b | NRKW | A66 | M | 16 | 206.55 | 8.47 | 32.02 | 1.19 | 0.89 |
| oo12_235b | NRKW | A66 | M | 16 | 155.39 | 2.99 | 29.56 | 2.42 | 0.61 |
| oo12_235b | NRKW | A66 | M | 16 | 170.48 | 2.91 | 15.98 | 2.10 | 0.33 |
| oo12_235b | NRKW | A66 | M | 16 | 174.35 | 4.21 | 39.26 | 0.17 | 0.74 |
| oo12_235b | NRKW | A66 | M | 16 | 150.87 | 3.37 | 34.22 | 0.26 | 0.45 |
| oo12_251m | SRKW | K33 | M | 11 | 121.08 | 7.24 | 57.16 | 2.87 | 0.98 |
| oo12_251m | SRKW | K33 | M | 11 | 103.45 | 3.32 | 48.07 | 2.69 | 0.77 |
| oo12_251m | SRKW | K33 | M | 11 | 108.44 | 5.89 | 30.64 | 0.62 | 0.96 |
| oo12_254m | SRKW | L95 | M | 16 | 97.97 | 4.17 | 21.95 | 0.24 | 0.78 |
| oo12_254m | SRKW | L95 | M | 16 | 349.02 | 5.10 | 9.66 | 2.09 | 0.61 |
| oo12_254m | SRKW | L95 | M | 16 | 121.73 | 2.81 | 8.60 | 2.88 | 0.82 |
| oo12_254m | SRKW | L95 | M | 16 | 107.47 | 5.17 | 9.21 | 2.51 | 0.84 |
| oo12_254m | SRKW | L95 | M | 16 | 108.33 | 2.23 | 13.27 | 1.13 | 0.49 |
| oo12_254m | SRKW | L95 | M | 16 | 178.80 | 3.97 | 41.20 | 1.35 | 0.89 |
| oo12_254m | SRKW | L95 | M | 16 | 121.39 | 2.68 | 21.68 | 2.93 | 0.52 |
| oo12_254m | SRKW | L95 | M | 16 | 132.21 | 2.90 | 33.60 | 1.09 | 0.70 |
| oo12_261m | SRKW | L84 | M | 22 | 55.55 | 2.05 | 17.45 | 2.02 | 0.73 |
| oo12_261m | SRKW | L84 | M | 22 | 89.51 | 3.38 | 28.56 | 2.48 | 0.77 |
| oo12_266m | SRKW | L91 | F | 17 | 97.30 | 3.87 | 21.70 | 2.89 | 0.67 |
| oo12_266m | SRKW | L91 | F | 17 | 89.09 | 3.06 | 16.58 | 3.01 | 0.48 |
| oo12_266m | SRKW | L91 | F | 17 | 121.53 | 4.00 | 13.93 | 2.32 | 0.87 |
| oo12_266m | SRKW | L91 | F | 17 | 134.75 | 5.89 | 24.56 | 0.90 | 0.76 |
| oo14_249m | SRKW | L113 | F | 5 | 124.16 | 4.00 | 28.72 | 1.18 | 0.40 |
| oo14_249m | SRKW | L113 | F | 5 | 77.19 | 4.23 | 46.57 | 1.51 | 0.80 |
| oo14_263m | SRKW | L85 | M | 23 | 187.77 | 3.75 | 33.78 | 0.80 | 0.68 |
| oo14_263m | SRKW | L85 | M | 23 | 196.89 | 8.94 | 21.18 | 1.58 | 0.75 |
| oo14_263m | SRKW | L85 | M | 23 | 211.70 | 5.23 | 45.34 | 3.13 | 0.75 |
| oo14_264m | SRKW | L91 | F | 19 | 138.53 | 2.94 | 56.48 | 2.47 | 0.40 |
| oo14_264m | SRKW | L91 | F | 19 | 155.12 | 6.92 | 79.60 | 2.31 | 0.83 |
| oo14_266m | SRKW | K35 | M | 12 | 93.49 | 7.07 | 63.27 | 2.40 | 0.85 |
| oo14_266m | SRKW | K35 | M | 12 | 88.09 | 5.07 | 41.17 | 2.94 | 0.71 |
| oo14_266m | SRKW | K35 | M | 12 | 90.51 | 1.95 | 39.56 | 2.32 | 0.61 |
| oo14_266m | SRKW | K35 | M | 12 | 156.30 | 5.16 | 37.28 | 1.30 | 0.69 |
| oo14_266m | SRKW | K35 | M | 12 | 121.77 | 3.84 | 55.69 | 2.81 | 0.81 |
| oo14_266m | SRKW | K35 | M | 12 | 157.65 | 5.40 | 80.26 | 3.06 | 0.65 |
| oo14_266m | SRKW | K35 | M | 12 | 39.52 | 2.35 | 9.00 | 0.24 | 0.43 |
| oo14_266m | SRKW | K35 | M | 12 | 139.10 | 3.82 | 37.65 | 2.36 | 0.83 |

Table S2. Comparison of effect levels for all models.

| **Model ID** | **Response** | **Effect 1** | **Effect 2** | **lsmean** | **s.e.** | **df** | **Lower.CL** | **Upper.CL** |
| --- | --- | --- | --- | --- | --- | --- | --- | --- |
| a | No. prey captured (offset by deployment duration) | *Population*  SRKW  NRKW  SRKW  NRKW | *Sex*  F  M  M  F | 1.34  1.85  2.25  2.45 | 0.321  0.232  0.195  0.181 | 49  49  49  49 | 0.51  1.25  1.74  1.98 | 2.17  2.45  2.75  2.92 |
| b | No. prey captured (offset by searching effort) | *Population*  SRKW  NRKW  SRKW  NRKW | *Sex*  F  M  M  F | 1.18  1.83  2.02  2.59 | 0.470  0.227  0.241  0.195 | 39  39  39  39 | -0.05  1.24  1.39  2.08 | 2.40  2.42  2.65  3.10 |
| c | Prop. time engaged in prey capture | *Population*  SRKW  NRKW  NRKW  SRKW | *Sex*  F  M  F  M | -2.54  -1.87  -1.49  -1.44 | 0.318  0.217  0.236  0.229 | 49  49  49  49 | -3.36  -2.43  -2.10  -2.03 | -1.71  -1.31  -0.88  -0.85 |
| d | Prop. time engaged in travel/resting | *Population*  SRKW  SRKW  NRKW  NRKW | *Sex*  M  F  M  F | -1.29  -0.93  -0.26  0.10 | 0.294  0.352  0.231  0.306 | 48  48  48  48 | -2.05  -1.85  -0.85  -0.69 | -0.53  -0.02  0.34  0.89 |
| e | Max. depth of prey capture dive | *Population*  NRKW  SRKW | -- | 3.86  4.31 | 0.129  0.162 | 40  40 | 3.56  3.93 | 4.16  4.69 |
| f | Bathymetry of prey capture dive | Sex  F  M | -- | 4.80  5.15 | 0.118  0.094 | 38  38 | 4.52  4.93 | 5.07  5.37 |
| g | No. prey captured by adult females | *Population*  SRKW  SRKW  NRKW  NRKW | *Calf*  Y  N  Y  N | -0.46  1.49  1.72  3.67 | 0.795  0.532  0.535  0.606 | 14  14  14  14 | -2.73  -0.03  0.20  1.94 | 1.81  3.01  3.25  5.40 |
| h | No. prey captured by adult males | *Population*  SRKW  NRKW  NRKW  SRKW | *Living mother*  N  Y  N  Y | 1.86  1.92  2.10  2.54 | 0.305  0.237  0.266  0.248 | 21  21  21  21 | 1.03  1.28  1.37  1.87 | 2.69  2.57  2.82  3.22 |
